# Single- and Cross-Modality Near Duplicate Image Pairs Detection via Spatial Transformer Comparing CNN

**DOI:** 10.3390/s21010255

**Published:** 2021-01-02

**Authors:** Yi Zhang, Shizhou Zhang, Ying Li, Yanning Zhang

**Affiliations:** 1 School of Computer Science, National Engineering Laboratory for Integrated Aero-Space-Ground-Ocean Big Data Application Technology, Shaanxi Provincial Key Laboratory of Speech & Image Information Processing, Northwestern Polytechnical University, Xi’an 710129, China; szzhang@nwpu.edu.cn (S.Z.); lybyp@nwpu.edu.cn (Y.L.); ynzhang@nwpu.edu.cn (Y.Z.); 2 School of Communication and Information Engineering, Xi’an University of Posts and Telecommunications, Xi’an 710121, China

**Keywords:** comparing CNN, spatial transformer network, near duplicate image pairs

## Abstract

Recently, both single modality and cross modality near-duplicate image detection tasks have received wide attention in the community of pattern recognition and computer vision. Existing deep neural networks-based methods have achieved remarkable performance in this task. However, most of the methods mainly focus on the learning of each image from the image pair, thus leading to less use of the information between the near duplicate image pairs to some extent. In this paper, to make more use of the correlations between image pairs, we propose a spatial transformer comparing convolutional neural network (CNN) model to compare near-duplicate image pairs. Specifically, we firstly propose a comparing CNN framework, which is equipped with a cross-stream to fully learn the correlation information between image pairs, while considering the features of each image. Furthermore, to deal with the local deformations led by cropping, translation, scaling, and non-rigid transformations, we additionally introduce a spatial transformer comparing CNN model by incorporating a spatial transformer module to the comparing CNN architecture. To demonstrate the effectiveness of the proposed method on both the single-modality and cross-modality (Optical-InfraRed) near-duplicate image pair detection tasks, we conduct extensive experiments on three popular benchmark datasets, namely CaliforniaND (ND means near duplicate), Mir-Flickr Near Duplicate, and TNO Multi-band Image Data Collection. The experimental results show that the proposed method can achieve superior performance compared with many state-of-the-art methods on both tasks.

## 1. Introduction

With the rapid development of portable multimedia sensors and mobile Internet technologies, the amount of multimedia data has been growing at a really fast speed. Thus, there are many near-duplicate images existing among the massive data. Near-duplicate images are typically considered as images containing the same scene or objects [1,2,3], but in various viewpoints, illumination conditions and camera settings etc, or images obtained through reediting of the original image [4], including but not limited to changing contrast, tone, cropping, rotating and watermarking. Automatic near-duplicate image pair detection by using computer vision and pattern recognition technology has been attracting wide attention recently, as it has great potential values in the application of image copyright violations detection, fake image detection, management of device hardware storage, and autonomous vehicle driving.

Conventional human-crafted local features-based approaches, such as the widely adopted scale-invariant feature transform (SIFT) [5], histograms of oriented gradients (HOG) [6] descriptors, obtain the image-level features through aggregating strategies, like vector of locally aggregated descriptors (VLAD) [7], Fisher Vector [8], etc. These methods suffer from the problem of complicated extraction steps and limited representation abilities. More recently, thanks to the great feature learning ability of convolutional neural network (CNN), researchers have resorted to CNN to deal with the near-duplicate image pair detection problem.

By using deep neural networks to represent images and combining different similarity measurement strategies to implement this task, CNN-based methods have achieved great performance. However, existing methods still show the following disadvantages. Vanilla CNN models incorporate many convolution and down-sampling/pooling layers to learn layered representations. Typically, higher layer features (low resolution) represent more semantic information which relates more to the content of the input image, while they contain less for pixel-level or even region-level information. In addition, conventional CNN models designed for image classification could not fully exploit the correlations between pairwise images. Most existing methods focus on the features of each image from the image pair, and they usually do not connect two images until the process of similarity measurement on the basis of these features, while few methods stack two images as an entire input and feed it into networks, which can fuse two images in very early processing phases but probably ignore the local information of each image to some extent.

**Figure 1:**
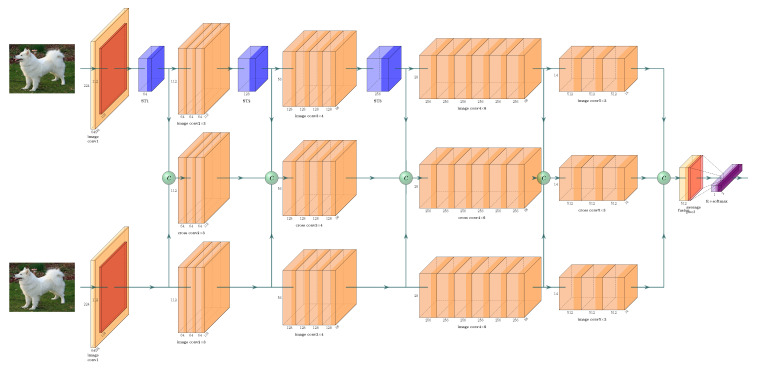
Illustration of spatial transformer-comparing convolutional neural network (ST-CCNN) architecture. The input images (showing in a slightly cropped image pair) are fed into two image streams (top and bottom), each of which consists of a convolutional block and four residual blocks. The cross stream (middle) shares the later residual blocks with the image streams. Feature maps of these three streams are fused by concatenation and convolutional layers to a vector into a fully connected layer and a Softmax layer.

To address these problems, in this paper, we propose a comparing CNN (CCNN)-based model to make more use of different resolution features to encode the correlations of image pairs and detect whether they are near duplicate pairs, as illustrated in Figure 1. This architecture focuses on learning to discriminate image pairs as near-duplicate or non-near-duplicate. Furthermore, to tackle the spatial variations, we introduce a spatial transformer (ST) module into the model, termed as ST-CCNN, to learn the features which are robust to cropping, translation, scaling, and non-rigid transformations. To demonstrate the effectiveness of the proposed method on both the single-modality and cross-modality (Optical-InfraRed) near-duplicate image pair detection task, we conduct extensive experiments on three popular benchmark datasets. The evaluation results show that the proposed method can achieve superior performances compared with many state-of-the-art methods.

In summary, the main contributions of this paper are threefold:

- We propose a CCNN model to the task of near-duplicate image pair detection, which makes more use of rich resolution features to encode the correlations between image pairs.
- We further propose the ST-CCNN model by introducing a spatial transformer module into the comparing CNN architecture, which can improve the robustness to variations, such as cropping, translation, scaling, and non-rigid transformations.
- Comprehensive experiments on both the single-modality and cross-modality (Optical-InfraRed) near-duplicate image pair detection tasks are conducted to verify the effectiveness of the proposed method.

The remainder of the paper is organized as follows: Section 2 reviews the literature on image comparing techniques. Section 3 elaborates the proposed model and the training method. Section 4 shows the experimental results and ablation studies. Section 5 discusses the proposed model. In Section 6, we draw the conclusion of the paper.

## 2. Related Work

To learn the correspondences and measure similarities within image pairs, from the perspective of image features extraction, existing work can normally be categorized into two-stage strategies and end-to-end strategies.

### 2.1. Two-Stage Strategies

Generally, the two-stage strategies are based on feature extraction of each image, then process on these features and output the comparative results. To obtain human-crafted local features, commonly, interest points or regions are detected through HOG [5] or Hessian-Affine [9], etc. Subsequently, SIFT [5], encoding the salient aspects of the image gradient in the neighborhood around feature points, is one of the most popular descriptors which are extracted on those interest point-based regions or densely sampled patches. Based on these, various descriptors, including but not limited to Fisher Vector [8], VLAD [7], PCA-SIFT [10], and Affine-SIFT [11], are proposed to the task and optimize it addressing kinds of problems. To learn the regional correlation with image pairs, Zhang and Chang [12] aligned interest points into an Attributed Relational Graph (ARG) that represents the compositional part-wise correlations in an image. However, the graph is low in efficiency due to the complex process of stochastic belief propagation. Xu et al. [13] encoded the spatial clue into the comparing by a spatially aligned pyramid. Zhang et al. [14] proposed an approach which is able to encode more spatial information into Bag-of-Visual-Word representation by geometry-preserving visual phrases (GVP) which can adequately adopted on large-scale databases. In the following comparing stage based on the features, Locality-Sensitive Hashing (LSH) [10] and Bag-of-Word (BoW) [11,15,16] are used to encode and aggregate local patches statistics. Zheng et al. [17] proposed to embed multiple binary features at indexing level to deal with the limitations of BoW.

As the widespread of deep learning, deep neural networks have achieved state-of-the-art performances in most computer vision tasks. Deep convolutional networks which can learn more high-level semantic information are introduced into the image features extraction for comparing. For content-based image comparing, Wan et al. [18], Babenko et al. [19] encode the features extracted by CNN into the global features. From global-level to patch-level, Gong et al. [20] proposed the Multi-scale Orderless Pooling (MOP) to represent image local information through aggregating CNN features at three scales, respectively. In [21,22], authors extracted CNN features on each object region which is detected by object proposal. Through these works, it could be observed that local descriptor and global descriptor can be suitable for specific types of data, respectively. Zheng et al. [23] adopted CNN on regional and global patches features extraction, to solve the contextual evidence of key-points mis-considered problem of traditional descriptors. They attempted to integrate discriminative cues from multiple contextual levels through probabilistic analysis and defined ‘true match’ as a pair of key-points corresponding to the same scene location on local, regional, and global levels. Different from using CNN as auxiliary cues to BoW, in the further similar work, Yan et al. [24] integrates SIFT and CNN co-equally in a complementary way to present an image in point-level, object level and scene-level. Subsequently, they compress combinations of several methods under different levels with simple PCA on commonly used benchmark datasets and achieve several state-of-the-art results. Babenko and Lempitsky [25] propose sum-pooled convolutional (SPoC) to obtain deep features with encoding the spatial clues of images and then apply PCA whiting and the $L_{2}$ measure to complete the near duplicate images detection. Based on hashing strategy, deep neural networks are also widely used with great effectiveness and efficiency [26,27], such as Multi-layer Orderless Fusion (MOF) [28] and convolutional neural network hashing (CNNH) [29].

### 2.2. End-To-End Strategies

Instead of features extraction and comparing them in two phases separately, Siamese networks [30,31] are introduced to process raw image pairs and output the comparative results directly. Thus CNNs are utilized to retain the model in an end-to-end way, by giving ‘yes or no’ labels between input images. By using Siamese networks, the work in Reference [32,33] achieved state-of-the-art results in their specific fields, which shows that end-to-end convolutional neural networks are well suited for comparing tasks. Focusing on spatial invariance, Jaderberg et al. [34] designed a spatial transformer network to deal with various transformations, including rigid and non-rigid distortion. Altwaijry et al. [31] equipped the ST modules on a Siamese-based network to complete aerial image matching, which shows a slight out-performance compared to the original Siamese-based network. Hashing-based methods also successfully applied the end-to-end strategy. Zhang et al. [35] proposed a learning framework to generate compact and bit-scalable hashing codes directly from raw images. Different from restricted Siamese network, the pseudo-Siamese uses the dual streams architecture but not sharing parameters. Through such a setup, pseudo-Siamese is able to provide more flexibility than restricted Siamese, while restricted Siamese is more efficient in training [36]. Furthermore, DeepRet [2,37] extends Siamese into triplet Siamese with the Triple Loss to capture the slight difference between images and applies region of interest (ROI) pooling to deeply learn the images.

Most approaches described above compare near-duplicate images mainly through learning similarity based on processing raw images separately. The images from one pair do not interact until they are broadcast into the decision layers at the late stage of the networks. Under such a strategy, the correlation between images, especially the strong correlation between near duplicate images, is not fully utilized [38]. For narrow baseline of image patch comparing [36], a network named 2-channel network allows the patch pair correlation to be learned at the very start of training. By fusing two images from the beginning and extracting deep features jointly, then directly comparing these features, the 2-channel architectures provides greater capacity of flexibility. However, its testing procedures on several databases, such as retrieval-based datasets, are expensive due to it requiring pairing and traversing all the images. For wider baseline, Zhang et al. [39] propose to apply 2-channel network on near duplicate image detection tasks. Specifically, to handle the non-rigid deformations and repetitive texture in image pairs, Revaud et al. [40] proposed images by stacking them into and generating subsets of ‘quadrants’ and ‘sub-quadrants’ by the DeepMatching to address the non-rigid motion which is hard to process for previous SIFT methods.

## 3. Methodology

To make more use of various resolution features and learn the image pair jointly, as shown in Figure 1, we apply a ST-CCNN with triple-stream-based model and ST modules to conduct near duplicate image comparing. Accordingly, in this section, we elaborate the proposed model in terms of triple-stream architecture and the ST modules, respectively.

### 3.1. Triple-Stream Framework

Given an image pair $\left(I_{l},I_{r}\right)$ as the input, the purpose of the models is to learn the similarity between its images and obtain a probability, which shows the possibility that the input image pair is a near duplicate (ND)/non-near duplicate (NND) pair. The process can be illustrated as

(1) $$P=S(I_{l},I_{r}),$$

where *P* refers to the probability, S(·) refers to the similarity learning, and $I_{l}$ and $I_{r}$ refer to left image and right image in the image pair. By building up an end-to-end network architecture and predicting the probability directly, the near duplicate image pair detection is converted into a classification task for image pairs. Depending on the input strategy of $I_{l}$ and $I_{r}$, existing architectures can be roughly categorized into 2-stream and 2-channel. The 2-stream framework that shares structures and parameters, namely Siamese network, feeds each image into each network stream, separately. Siamese is one of the most commonly used frameworks in the field of tasks processing image pairs, and it performs greatly in image comparing tasks. In the task of comparing, Siamese mainly utilize the stream equipped network as feature extraction and compare these features in decision layers. Based on these frameworks, there is hardly interactive processing before the latter comparing stage. Different from separately processing images, 2-channel framework have neglected the extraction of each single image features and stack $I_{l}$ and $I_{r}$ as one input into a network. Though the framework using this strategy can learn the correlation between images earlier than the 2-stream framework, its neglect of feature extraction of each image tends to lead to a missing consideration of low level or local features, especially on the network built on Vanilla CNN. In terms of patch comparing, 2-channel framework performs well [36]. However, when it is directly applied to wider scenario [39], it only performs well for images with low difficulty, such as images that are very visually similar, while, when images involve local correlation, such as replacement, viewpoints change, etc., this framework is hardly to accurately detect those challenging near duplicate pairs.

As shown in Figure 1, ST-CCNN processes image pairs using a triple-stream-based network. Among these streams, $I_{l}$ and $I_{r}$ are resized to 224 × 224 × 3 and fed into two image streams, the top and the bottom streams in Figure 1, respectively. The image streams share the backbone and parameters. Each image stream is composed of five convolutional sets, of which the first set is a combination of a single convolution layer, a Rectified Linear Unit (ReLU) layer and a max-pooling layer. The remaining four sets are combinations of residual convolutional blocks. Each convolutional set includes numbers of 3, 4, 6, and 3 residual blocks, and each residual block is a standard basic residual block, mainly consisting of two convolutional layers. The structure of the cross stream, which lies in the middle of two image streams in Figure 1, shares the later four convolutional sets with image stream. The input of each set is the combination of feature maps from each image stream and the cross stream itself on the same stage (the same ID of the convolutional sets). In order to provide greater capacity of feature extraction, these feature maps are combined through concatenation and a single convolutional layer followed by a ReLU layer. The cross stream can embed features from each image on multiple scales gradually and jointly, thus representing the potential displacement of relevant regions within the image pair. Considering the displacements those regions within ND pairs are usually limited, we apply each convolutional layer in the later four convolutional sets with unique kernel size of 3×3. In this way, the triple-stream network allows capturing potentially local and global correspondences by gradually fusion multi-scale features and residual learning in encoding the image pair into the one-dimension vector for classification.

### 3.2. Spatial Transformer

The changes between images in an ND pair usually include spatial transformations but without specific labeling on these changes, such as translation, rotation, cropping, etc. Vanilla CNNs achieve translation invariance to a certain extent through the max-pooling layers, that is, if the regions/objects transform within the pooling unit, the activation values can be identical, thus achieving certain invariance. However, the pooling units are generally with small sizes, commonly 2×2, which imposes an obvious limitation on the spatial invariance of Vanilla CNN. The introduction of triple-stream structure can solve multi-range displacement by carrying out the stack of multi-scale feature maps from each image stream. However, for scaling, rotation, and several non-rigid transformations, Vanilla CNN-based networks are difficult to cope with. Therefore, we introduce the ST modules to maintain the robustness of the model to various transformations of regions/objects within the image pairs.

**Figure 2:**
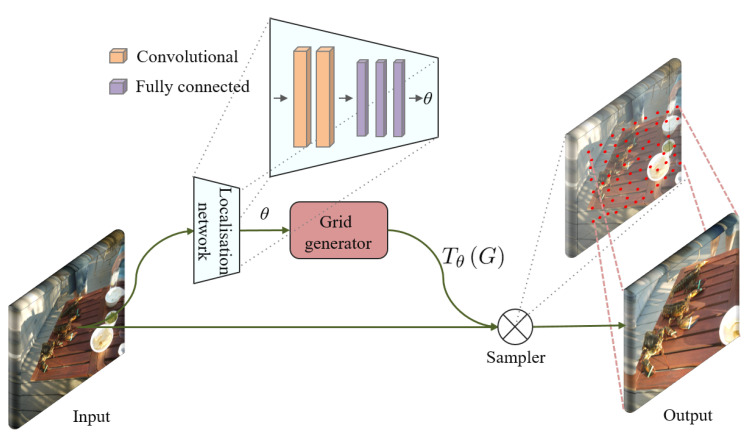
Illustration of a spatial transformer module architecture. The input feature maps *X* is fed into a localization network, which regresses the transformation parameters θ. The regular spatial grid *G* over output is transformed to the sampling grid $T_{\theta }\left(G\right)$ which is applied to input to generate the warped output feature map $X^{st}$.

The ST module is with a self-adaptive mechanism, which mainly consists of three components, as shown in Figure 2: the localization network returns the spatial transformer parameter θ of the input feature maps; and then the grid generator uses this parameter to generate a sampling grid $T_{\theta }\left(G\right)$; finally, according to this grid, the differentiable sampling kernel uses the grid $T_{\theta }\left(G\right)$ to sample the input feature maps, and then the output feature maps with spatial transformed is obtained. The first component, localization network, consists of two sets of a convolutional layer followed by a max-pooling layer, also a ReLU layer and three fully connected layers. The input to the localization network is the feature maps $X\in R^{H\times W\times C}$, and the output is the localization parameter $\theta =f_{loc}\left(X\right)$, where $f_{loc}\left(\cdot \right)$ means the regression of localization network. This parameter reflects the mapping of coordinate relationship between input *X* and the expected spatial transformed feature maps $X^{st}\in R^{H\times W\times C}$. The size of the parameter is set to a classic 2D affine transformation with dimension of 2×3, which can deal with cropping, translation, scaling and rotation. Based on this, the second component grid generator performs matrix operations with all coordinates in ordinary grid $G=\left\{G_{i}\right\},G_{i}=\left(x_{i}^{st},y_{i}^{st}\right)$ in $X^{st}$ as independent variables and with θ as the parameter, so as to obtain the corresponding coordinate points grid $T_{\theta }\left(G\right)=\left\{G_{i}^{src}\right\},G_{i}^{src}=\left(x_{i}^{src},y_{i}^{src}\right)$ of each coordinate in $X^{st}$ at the input *X*. This operation can be represented as follows:

(2) $$\left(\begin{array}{c} x_{i}^{src}\\ y_{i}^{src} \end{array}\right)=\theta \left(\begin{array}{c} x_{i}^{st}\\ y_{i}^{st}\\ 1 \end{array}\right)=\left[\begin{array}{ccc} \theta_{11} & \theta_{12} & \theta_{13}\\ \theta_{13} & \theta_{22} & \theta_{23} \end{array}\right]=\left(\begin{array}{c} x_{i}^{st}\\ y_{i}^{st}\\ 1 \end{array}\right),$$

where *i* means the index of all coordinates of the feature maps $X^{st}$, $\left(x_{i}^{src},y_{i}^{src}\right)$ refers to the coordinate values mapping to the *i*-th coordinate $\left(x_{i}^{st},y_{i}^{st}\right)$ in $X^{st}$, that is, the source coordinate of *i*-th coordinate in $X^{st}$ is $\left(x_{i}^{src},y_{i}^{src}\right)$ in the input *X*. Then, the third component differentiable sampling kernel takes *X* and $T_{\theta }\left(G\right)$ as input to calculate each pixel value in $X^{st}$. Considering the spatial transformation information of ND pairs is mainly involved in high resolution features, such as displacement, pose change, and cropping in small range, we deploy the ST modules on the shallow convolutional sets which with smaller respective fields than deeper sets. In order to avoid that the features extraction of each image stream tend to be equal, we deployed ST modules on only one of the image streams, which will be clarified in the subsequent Section 4.4.3

### 3.3. Loss Function

The feature maps obtained by the three streams are mapped to a one dimension vector in length of 2, by concatenating and average pooling. After a Softmax layer, all values in the vector are always in the range [0,1], and add up to 1, thus forming a probability distribution. According to the image classification strategy, we consider that the index of the larger value represents the category of the input image pair, that is, index 1/0 corresponds to ND/NND. The training is supervised by the label l∈{0,1} of each image pair $\left(I_{l},I_{r}\right)$ with binary cross entropy loss function, which can be presented as follows:

(3) $$Loss\left(p_{i},l_{i}\right)=-w_{i}\left[l_{i}logp_{i}+\left(1-l_{i}\right)log\left(1-p_{i}\right)\right],$$

where *i* refers to the *i*-th image pair in the training dataset, $p_{i}$ refers to the probability that the input image pair is predicted as an ND pair, $l_{i}$ refers to the real label of input image pair is an ND pair, and $w_{i}$ refers to the weight of each batch. Considering the ST-CCNN is an asymmetric network, we cover all the training and testing image pairs with their left and right flips, and we completely random input them to the networks.

## 4. Experiments

### 4.1. Datasets and Settings

We implement our model on three benchmark datasets: CaliforniaND [3] and Mir-Flickr Near Duplicate (MFND) [4] for single-modality near duplicate image pairs comparing, while TNO Multi-band Image Data Collection (TNO) [41] for cross-modality near duplicate image pairs comparing.

**Figure 3:**
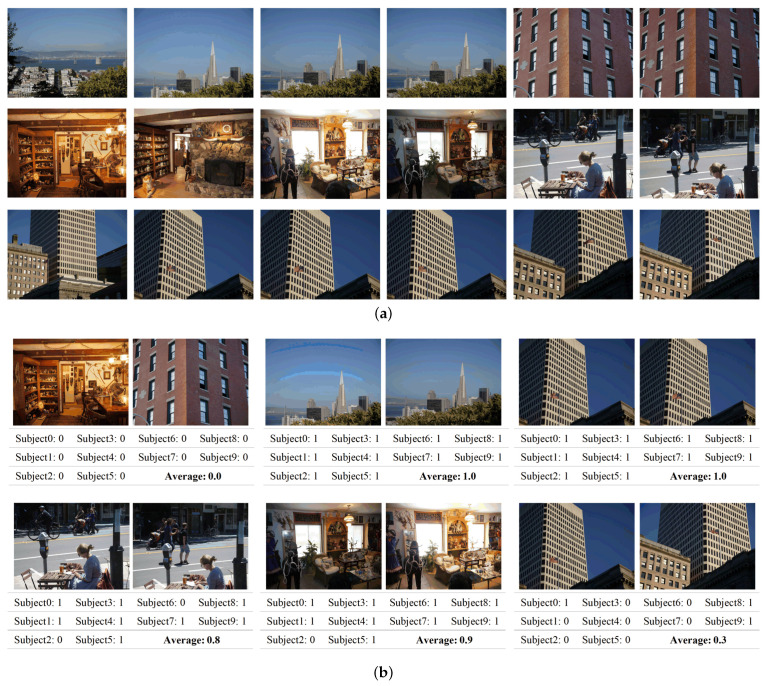
Illustration of examples from CaliforniaND (ND = near duplicate). (**a**) Raw photos in CaliforniaND. (**b**) Image pairs with their binary annotations and the average probability to be a near duplicate image pair. In the first row, three samples are given by all subjects with the same binary label, which are obvious near duplicate/non-duplicate image pairs. The second row shows pairs that are not extremely visually similar. The changes between pairs, which are considered as near duplicate averagely, include rotation and translation of views, target changes, and camera exposure setting changes.

*CaliforniaND* is a dataset containing 701 photographs taken directly from a real personal travel photo collection taken by one photographer. As shown in Figure 3a, the original images in this dataset are usually consecutive shots (including burst shots, moving background shots, panoramic shots, etc.) of similar scenes during a short time period. It contains many difficult cases and types of near duplicate pairs. To deal with the inevitable ambiguity presented by near duplicate pairs, this dataset is annotated by giving binary decisions from 10 subjects, including the photographer himself. As illustrated in Figure 3b, these annotations correlation matrices are combined and averaged into a non-binary ground truth, presenting the probability that the observers might consider a pair of images are near duplicate. Each image considered as a near duplicate can have change of target or background, different camera parameter settings, image noise, color environment, and resolution, and other factors will affect the image quality. Our experiments are implemented on the average matrices of 10 subjects. This dataset is relatively challenging due to the fact that it contains many non-identical ND pairs of real-world scenes [1].

**Figure 4:**
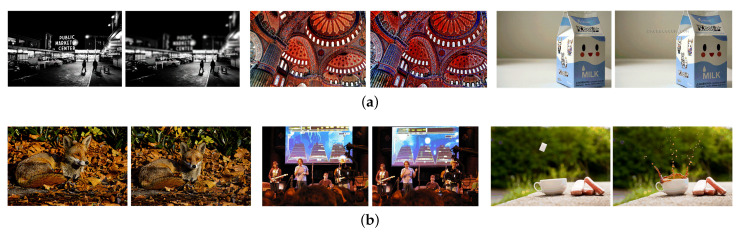
Illustration of near duplicate image pair samples from Mir-Flickr Near Duplicate (MFND). (**a**) Pairs from MFND-identical near-duplicate (IND). From left to right, the changes between near duplicate pairs are focus change, scale change, color tune change, border, and watermark addition. These pairs are extremely visually similar, and there is no displacement or posture change of the objects or background in the images. (**b**) Pairs from MFND-non-identical near-duplicates (NIND).

*MFND* is a benchmark near duplicate detection based on the pre-existing MIR-FLICKR collection. As shown in Figure 4 Image pairs are labeled as duplicates, identical near-duplicate (IND), and non-identical near-duplicates (NIND). Duplicate images are defined as those are the same size and have the same value in each pixel. IND pairs appear to have been derived from a common precursor via manipulation. NIND pairs are non-duplicate pairs, but which nonetheless have a striking visual similarity. From the perspective of human vision, the difficulty of comparing these pairs and giving binary decisions is that NIND is more difficult than IND, and IND is more difficult than duplicates. Following other methods, our experiments on this dataset are mainly carried out on subset MFND-IND and MFND-ALL, where ALL indicates IND and NIND.

**Figure 5:**
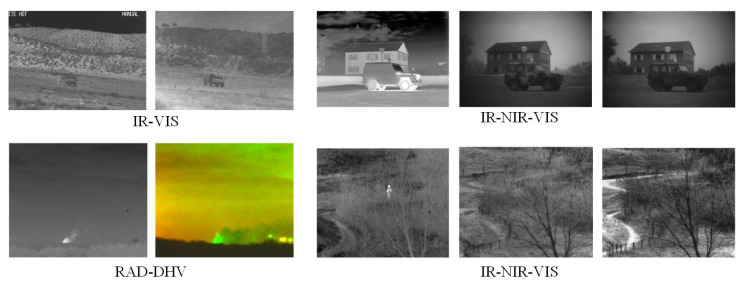
Illustration of examples in TNO. In different imaging modalities, the foreground and background are emphasized according to their temperature states. For instance, the texture features of the object are not clear in the night visible image (upper right), or even the objects are not obvious.

The *TNO* consists of multi-spectral (intensified visible (390–700 nm), near-infrared (700–1000 nm), and long-wave infrared or thermal (8–12 m)) nighttime imagery of different military relevant scenarios, registered with different multi-band camera systems, including Athena, dark hard vitreous (DHV), free electron laser (FEL), and TRI-band Color Low-light OBServation(TRICLOBS). We fed image-pairs from identical scenes as near duplicate pairs into proposed models. As shown in Figure 5, cross modality images captured on identical scenes showing identical targets (e.g., people, vehicles) in different (e.g., rural, urban) backgrounds.

To observe and verify the effectiveness of the proposed models, in Section 4.3, we compare our proposed networks with state-of-the-art methods. In addition, we organize sufficient ablation study on the proposed network in Section 4.4. These experiments are conducted with the platform of PyTorch (version 1.0.1) under the Ubuntu 16.04 operation system on a workstation with Intel Xeon(R) E5-2640 v4 @2.40 GHz and NVIDIA GTX 1080Ti GPU, with batch size of 8 image pairs, and learning rate at $5\times 10^{-4}$. For each dataset, we select all ND pairs (average metric with threshold = 0.5 in CaliforniaND) and randomly select NND pairs with the number of approximately equal to the number of ND pairs. We randomly divide experimental data from each dataset into a train set and test set according to about 3:1. According to the data size of the datasets, we adopt the random stochastic data augmentation, that is, according to a random augmentation probability, input $I_{l}$ and $I_{r}$ in were randomly performed identical transformation in the following modes when loading data of each batch, including horizontal flipping, vertical flipping, and rotation of $90^{\circ }$, $180^{\circ }$, and $270^{\circ }$. In all implementations, we use two backbones, the standard Visual Geometry Group Network-16 (VGG16) and ResNet34. The parameters for ST modules equipped are shown in Table 1 (where FC refers to fully connected layers).

### 4.2. Evaluation Metrics

We evaluate the prediction results with ground truth labels based on confusion matrix. Specifically, we use Accuracy, Recall, Precision, F1-score, and area under the ROC (AUROC), where ROC means receiver operating characteristic. For the proposed model, the output from Softmax layer of each image pair is a vector in size of 1×2. The sum of two values is 1.0 and, respectively, presenting the probabilities that the model predicts the input image pair belongs category-0 (non-duplicate) or category-1 (near-duplicate). Therefore, according to the common principle of image classification, we consider the index of the maximum value in this vector as the predicted binary label (equivalent to threshold = 0.5). Thus, in the case that all datasets used are completely manually labeled, based on the predicted binary labels and ground truth labels, we can obtain the complete confusion matrix items, namely true positive (TP), true negative (TN), false positive (FP), and false negative(FN). Based on this, the following measurements calculate as:

(4) $$Accuracy=\frac{TP+TN}{TP+TN+FP+FN},$$

(5) $$Precision=\frac{TP}{TP+FP},$$

(6) $$Recall=\frac{TP}{TP+FN},$$

(7) $$F1=2\times \frac{Precision\times Recall}{Precision+Recall},$$

where Precision refers to the proportion of the real positive to all the ‘positive’ predicted by the model, while Recall refers to the proportion of the model predicted ‘positive’ to all real positive samples. These two measurements are a pair of contradictory measurements; thus, the F1-score is considered as with more comprehensively measurement ability.

The evaluation above is based on the predicted binary labels. Furthermore, based on predicated probability, we use ROC and the area under it, which are one of the most widely used measures in machine learning to evaluate classification ability and robustness. ROC curve is a curve reflecting the relationship between sensitivity and specificity. Its x-axis is 1-specificity, also known as false positive rate (FPR). Its y-axis is sensitivity, also known as true positive rate (TPR). Thus, according to the curve, the entire plot is divided into two parts. The area under the curve (AUC) represents the prediction ability. The higher AUC value is, the superior the model prediction ability will be. The closer the curve is to the upper left corner (smaller x, larger y), the more accurate the prediction will be.

### 4.3. Result Comparison

To prove the effectiveness of the proposed model, we compare the proposed ST-CCNN with state-of-the-art methods. These methods include (1) 2-channel network [39], which stacks images from an image pair as one input and fed into neural networks, based on VGG16 and ResNet34; (2) SPoC [2] based on VGG16 and pretrained on Place205 [42] (SP-VGG16-PL), SPoC based on VGG19 and pretrained on ImageNet [43] (SP-VGG19IN), SPoC based on VGG16 and pretrained on Hybrid dataset (SP-VGG16-HY) where Hybrid is Place205&ImageNet, ResNet101 and pretrained on ImageNet (ResNet101-IN) and ResNet152-IN [2] utilize backbones pre-trained on Place205, ImageNet, Hybrid(showing in names by PL, IN, H, respectively) to the SPoC, which extract features from the pre-trained neural networks and spatially aggregated using sum pooling, and then measure the features by $L_{2}$ normalization after PCA whitening and compression; and (3) DeepRet500-L and DeepRet800-L [2] (where L refers to fine-tuning on Landmarks dataset) firstly conduct ROI pooling from images and feed the image triples separately using triplet loss on this basis. The advantage of this method is that it is able to apply a compact global image representation and improve the accuracy by using triplet loss for the slight changes between images. The AUROC results comparison were summarized as shown in Table 2 and Table 3, on single-modality datasets and cross-modality dataset, respectively. The best AUROC values are highlighted with bold and red, while green and blue refer to the second-best and the third-best, respectively.

#### 4.3.1. Single-Modality Results

It can be observed in Table 2 that, in the challenging dataset CaliforniaND, our network performs superior than state-of-the-art methods obviously. The networks of 2-channel perform obviously lower than other comparison methods, due to the simple strategy of concatenating images before input to the network. Though this strategy can process images jointly, because of its neglect of single image features extraction, especially the low resolution local features, it is hard to correctly detect the ND pairs with objects/background change and visually similar NND pairs. For SPoC frameworks based on VGG16-PL, VGG16-HY, and VGG19-IN, the networks based on VGG16 achieve AUROC around 0.900+, while VGG19 achieves 0.887. It indicates that, for challenging pairs, a deeper network does not lead to better performance. With the introduction of residual learning by shortcuts and deep network structures, the ResNet-based networks improve performance by more than 2%. However, the 512-layer network is not superior than the 101-layer network on this dataset, which indicates that a deeper network is not necessarily a better result under the same network architecture. By introducing the triplet loss focusing on slight difference and ROI pooling, the DeepRet based on ResNet101 performs similarly in CaliforniaND with SPoC methods based on ResNets. It indicates that the use of ROI pooling and the region proposal network (RPN) in the test phase is not sufficient to mine more of the changes that occur within image pairs. Our proposed ST-CCNN reaches the top and the second top AUROC on CaliforniaND, based on ResNet34 and VGG16, respectively. This may be because the introduction of cross stream and ST can deal with the displacement, rotation, scaling, and view change of foreground or background targets with image pairs, which provides possibilities for superior classification ability of the networks.

In MFND, we will discuss MFND-IND and MFND-ALL, respectively. In MFND-IND, a subset that is considered relatively simpler than other datasets [1,2], which can be observed in Figure 4a. The performance gaps among methods are not significant, and the performance is gradually improved as the networks going deeper also the structures becoming more complex. On this subset, DeepRet800 reaches the top performance at 0.996, followed by our ST-CCNN and DeepRet500, which are only 0.002 below. In addition, our ST-CCNN is based on ResNet34, which is much less layers than the backbone ResNet101 of DeepRet500. In MFND-ALL, which includes more challenging pairs, our ST-CCNN reaches the top AUROC. Generally, the overall performance distribution of all methods is similar to that of CaliforniaND, except that the improvement of SPoC backbones from VGGs to ResNets is more obvious. In addition, the structure of DeepRet shows significant advantages than in CaliforniaND. The advantage is that the design of ROI pooling and triplet loss is particularly suitable for MFND-ALL data, including some content changes of the targets, spatial changes (less than pairs in CaliforniaND) that can be captured by the triplet loss which is designed specifically for the slight differences, and ROI pooling also provides capacity for interesting regions concerns. Compared with VGGs-based networks, our ST-CCNN-VGG16 exceeds the others by more than 4%. While based on ResNet34, our ST-CCNN achieves the top AUROC at 0.992, which indicates that our model also performs well on the data with slight spatial variations.

#### 4.3.2. Cross-Modality Results

As shown in Table 3, all methods AUROC values do not exceed 0.80. The difficulties of ND detection on TNO lie in the following two aspects: (1) the limited size of the dataset; and (2), for different wave length on the identical scene imaging, these images mainly correspond in the correlation of various saliency regions, including identical targets of various visual views, such as people, vehicles, buildings, and paths. The first limitation of dataset size is reflected in all comparing methods. In particular, under the same architecture, the AUROC of ResNet152 pre-trained is lower than ResNet101, also the VGG19 is lower than that of VGG16, which may be caused by over-fitting problem. The later limitation is particularly apparent for networks that mainly use global features extraction, where the performance of the 2-channel and SPoc frameworks is obviously lower than other networks. DeepRet and our ST-CCNN have significant advantages due to their saliency region focusing design applied to these networks, and our ST-CCNN shows more effectiveness and reaches the top AUROC at 0.782.

### 4.4. Ablation Study

We implement ablation experiments on single-modality and cross-modality datasets, CaliforniaND and TNO, respectively. Based on the backbone of VGG16 and ResNet34, the ablation analysis of triple-stream, ST, and asymmetric ST structures are conducted, including quantitative comparison and visualization.

#### 4.4.1. Effectiveness of Triple Stream Structures

We implement comparing CNN in with/without the triple stream, presenting as CCNN-w-cross (CCNN with cross-stream) and CCNN-o-cross (CCNN without cross-stream to prove the effectiveness of our triple stream frameworks. As shown in the quantitative comparison Table 4, the addition of the triple stream can obviously improve the performance in F1-score and AUROC. Specifically, on VGG16 backbone, benefits from its great ability of image representation, the CCNN-o-cross network achieves 0.910 in recall, which means a good ND detection ability. However, its precision is 0.811, far lower than recall, which indicates that a certain number of pairs that are predicted to be ND are truly NND. By introducing the triple stream, CCNN-w-cross can slightly improve all measurements than CCNN-o-cross network but still shows a large difference in precision and recall. The possible reason is that its stacking of convolutional and pooling layers in each VGG16 streams would neglect the discriminative information of NND in the image features extraction process. Similarly, CCNN-o-cross network based on ResNet34 still has the problem of recall far exceeding precision. While the CCNN-w-cross based on ResNet34 solves this problem well, which indicates that the residual learning combined with the gradually stacking of multi-scale feature maps from dual streams in cross stream can properly retain both ND and NND discriminative information, providing the capacity for the network to accurately predict the category of the input image pair.

For cross-modality data, showing in Table 5, compared with dual stream networks, the introduction of cross stream can obviously improve the scores of all measurements, especially when ResNet34 is applied as the backbone. To be specific, the most relatively obvious improvement is on precision scores, which may be due to the introduction of cross stream which can provide more discriminative information as NND pairs when image stream feature maps tend to identify an NND as an ND. However, the recall scores are higher than the precision scores in all dual stream and triple stream networks.

**Figure 6:**
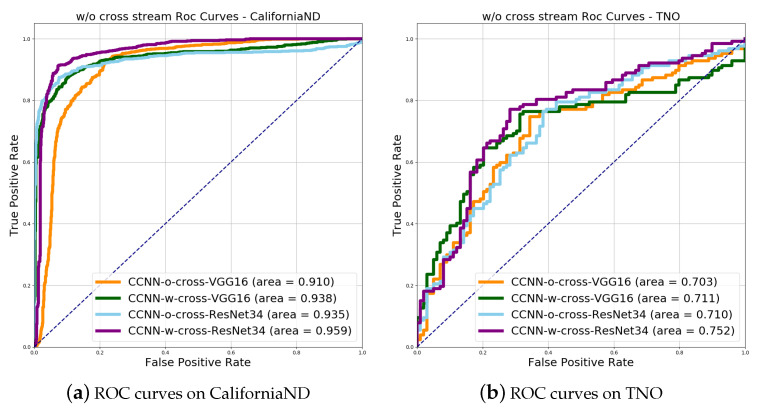
Illustration of ROC curves of networks with/without cross stream on single-modality and cross-modality. In both scenarios, CCNN-w-cross networks obviously perform better than CCNN-o-cross networks.

It can be observed in Figure 6 that, among two-stream and triple-stream networks based on VGG16 and ResNet34, the classification ability of CCNN-o-cross based on VGG16 is obviously lower than other networks, while the classification ability of CCNN-w-cross based on ResNet34 is relatively the best on both single-modality and cross-modality, with the largest area and the closest to the upper left corner. The difference between the two modality situations is mainly reflected in the CCNN-o-cross network based on ResNet34. In single-modality, the ResNet with stronger image representing capability shows its effectiveness. However, in cross-modality, residual learning still hardly solves the problems that VGG networks find hard to deal with.

**Figure 7:**
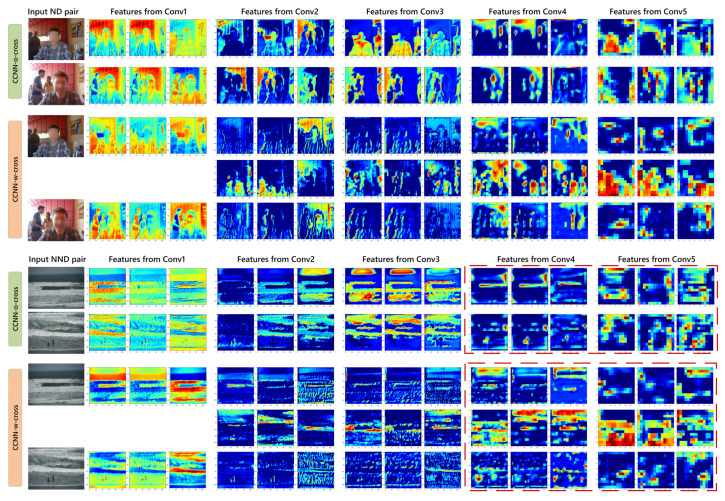
Visualization of the first three slices feature maps on each convolutional set of networks with/without triple stream. As highlighted in the red dashed boxes, for visually similar non-near duplicate (NND) pair, which is easily predicted as near duplicate (ND) by CCNN-o-cross, CCNN-w-cross is able to capture more discriminative information of visually similar NND.

To better observe the network, in Figure 7, we visualize the output feature maps of each convolutional sets (represented as ‘Conv1–Conv5’) of a challenging ND pair and NND pair. The ND pair is within the foreground and background objects moving, while the NND pair is visually similar. It can be observed that, for the ND pair, CCNN-w-cross makes the feature maps in two image streams closer than CCNN-o-cross. For the NND pair, the feature maps of each image in deep layers of CCNN-o-cross are basically similar. This corresponds to the previous quantitative comparison that its recall is much higher than precision, while the feature maps in CCNN-w-cross image streams show greater discrimination than CCNN-o-cross.

#### 4.4.2. Effectiveness of Spatial Transformers

We implement ST-CCNN in with/without ST modules, presenting as CCNN versus ST-CCNN, based on VGG16 and ResNet34 on the challenging dataset CaliforniaND. The quantitative comparison in Table 6 indicates that, based on both backbones, the introduction of ST modules can significantly improve precision, (increase by 0.102 on VGG16 and 0.050 on ResNet34). For VGG16, ST modules tackle the problem of a high false alarm rate which the triple stream hardly deals with. Based on ResNet34, ST modules can help with the increase of 0.054 of recall. Thus, ST-CCNN based on ResNet34 achieves best among all comparing ablation models with F1-score at 0.958 and AUROC at 0.990.

The introduction of ST modules effectively deals with the false alarm problem that cross stream is difficult to solve, as shown in Table 7, based on both backbones, ST-CCNN’s precision scores are higher than recall scores. However, the recall of ST-CCNN based on VGG16 is obviously reduced, which may be due to the limited data and the VGG structure of continuously pooling. In addition, there is barely spatial transformations of the visually different identical targets from ND pairs from TNO. For the above reasons, ST modules lack guidance, which improves the precision of ND pairs, while reducing the detection rate at the same time. The use of ResNet solves this problem, with the injection of residual learning by shortcuts, retains high resolution features information appropriately, provides sufficient courage for adaptive self learning, and enables the recall and precision to maintain higher scores, thus achieving the top F1 score and AUROC value.

**Figure 8:**
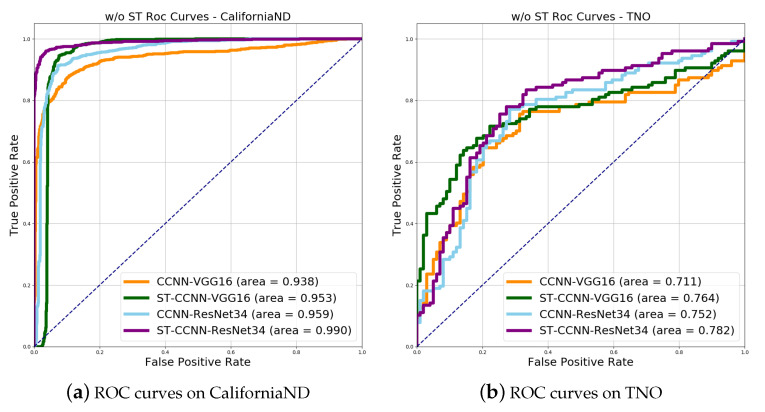
Illustration of ROC curves of networks with/without ST modules on single-modality and cross-modality. In both scenarios, ST-CCNN-based networks perform better than networks without ST modules.

As shown in Figure 8, among networks with/without ST modules, under single-modality, the ROC curve of ST-CCNN based on ResNet is the closest to upper left corner and with the highest value of AUROC. The ST-CCNN based on VGG16 and CCNN based on ResNet34 achieve similar AUROC. The ST-CCNN based on VGG16 has higher TPR when they range similar FPR, which means its ND detection capability is higher than the CCNN based on ResNet34, while the probabilities predicted by CCNN based on ResNet34 are distributed near ground truth label {0, 1}, which enables it to achieve a similar AUROC at a slightly lower F1-score with ST-CCNN based on VGG16. While under cross-modality, though the ST-CCNN-ResNet34 reaches the highest AUROC value, it is not obviously closer to the upper left corner than other curves because ST-CCNN-VGG16 draws closest to y-axis, indicating a higher precision. Generally, ST-CCNNs present obvious advantages compared to networks without ST modules.

**Figure 9:**
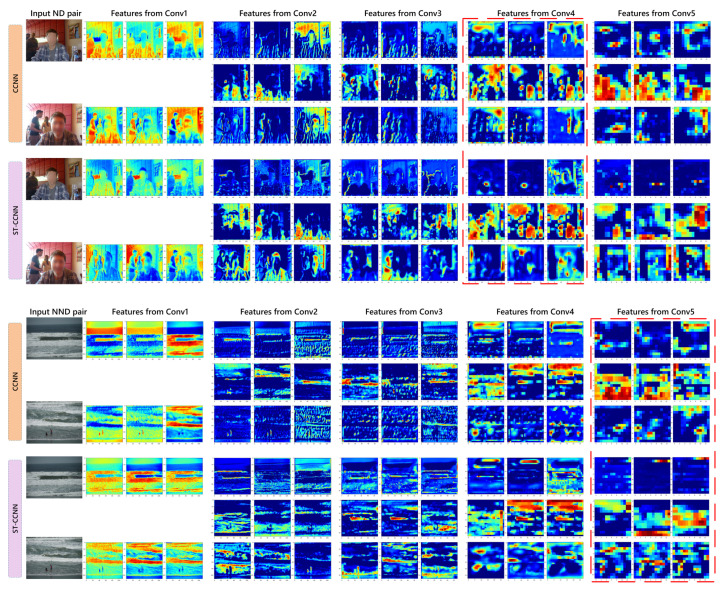
Visualization of the first three slices feature maps on each convolutional set of networks with/without the ST modules. For the ND pair, as shown highlighted by red dashed box, cross stream feature maps of ST-CCNN are more concentrated in the identical regions with slight displacements and zooming of the ND pair. For the NND pair, the feature maps in image streams show more obvious discrimination and the feature maps slices in the cross stream capture more saliency regions of each image in the NND pair, which can provide greater capacity for accurate decisions on challenging pairs.

The visualization of feature maps of networks with/without ST modules are illustrated in Figure 9. It can be observed that the changes in the ND pair showing is that the foreground male portrait has a slight pose change and zooming, and the background objects move and change. Compared with CCNN, as shown highlighted by a red dashed box, ST-CCNN performs proper transformation on the foreground object, making it more similar to the pose in the other input image. Meanwhile, the feature maps in the cross stream are more concentrated in the identical regions of the input images. For the visual similar challenging NND pair shown in the illustration, the deeper feature maps show that CCNN is able to capture discriminative information for non-duplicate, while the feature maps of image streams of ST-CCNN shows discrimination more obviously. The feature maps slices in the cross stream of ST-CCNN focused on the saliency regions of each image in the NND pair, which provides sufficient information for accurate classification of whether the pair is ND or NND.

#### 4.4.3. Effectiveness of Asymmetric ST Structure

**Figure 10:**
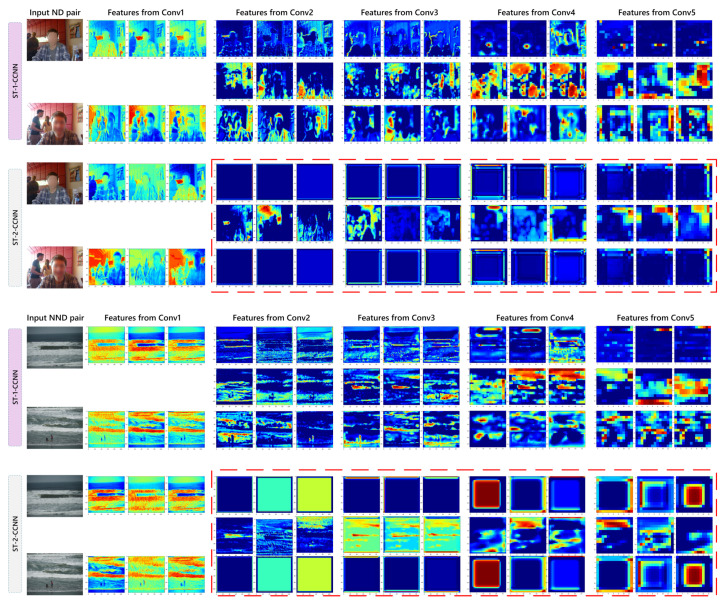
Visualization of the first three slices feature maps on each convolutional set of networks with ST modules on single/dual image stream. The dual here represents the CCNN equipped with parallel ST modules not sharing parameters.

We implement ST-CCNN with a single image stream equipped with ST modules (ST-1-CCNN) and two image streams equipped with ST modules (ST-2-CCNN). In the experiments, it can be observed that, on ST-2-CCNN, whether or not the parallel ST modules share parameters, the recall scores are extremely high, while the precision scores are much lower than the recall. It indicates that the dual ST modules lead to a high false alarm rate. We visualized examples in Figure 10 to discuss it.

As illustrated in Figure 10, we present the feature maps of single and dual ST-CCNN models. In order to simplify the expression, the ST-2-CCNN represents the ST-2-CCNN not sharing ST modules parameter, which performs relatively better in quantitative measurements among ST-2-CCNNs. It can be observed that, when ST-CCNN is dually equipped with ST modules, regardless of whether the input is an ND or NND pair, the feature maps of image streams tend to be equal. It leads to the fact that the effective feature extractions are mainly on the cross stream. However, the decision layers operate on the fusion of feature maps from image streams and cross stream, which makes the accuracy of ST-2-CCNN probably lower than the preliminary 2-channel architecture. The possible reason is that the training process of ST self-adaptive mechanism requires effective and sufficient guidance, while, in this task, the ground truth is a simple binary label. In the gradient backward process, if both image streams involve self-adaptive parameters, insufficient guidance may lead to the activation tending to be zero and being the same as each other, which reduces the binary classification precision.

## 5. Discussion

The existing methods rarely process image pairs jointly with well consideration of both low- and high- level features which can provide sufficient information for accurate near duplicate detection. In addition, the ND pairs are usually with spatial transformation of identical foreground or background objects, which has not been explicitly labeled. Based on these, we propose to perform an ST-CCNN to conduct the near duplicate image pairs detection task.

By comparing with state-of-the-art methods on AUROC, which can well measure the binary classification abilities of models, it proves the effectiveness of our proposed network, especially on the more challenging datasets. Such challenging data is mainly reflected in the identical targets displacement, scaling, rotation, etc., of the foreground or background, which leads to obvious shortcomings in existing methods. The advantages of our proposed model may benefit from the introduction of the cross stream to fuse low- and high- level features gradually, while considering the near range displacements. Moreover, the potential correlation within the image pairs is learned by the self-adaptive ST-modules to strengthen the model discrimination capturing abilities on ND pairs with spatial changes and visually similar NND pairs, so as to make the model superior to other models.

It is worth mentioning that the ST modules increase a certain number of parameters; thus, it requires sufficient data support in the process of self-adaptive learning, which has obvious limitations on its flexibility and learning capability. How to solve this problem in future research is worth addressing. The possible direction could include intensive learning, weakly supervised learning, in recent years, and so on.

## 6. Conclusions

In this work, an ST-CCNN model is proposed to conduct the single-modality and cross-modality near duplicate image pairs detection. Specifically, a triple-stream network is applied to fuse multi-scale features gradually, which allows the model to process images from a pair jointly and provides more capacity for learning the correlation between images. In addition, the self-adaptive ST modules are introduced to address the spatial transformations which are not explicitly labeled. Our model achieves superior performance on three benchmark datasets compared with state-of-the-art methods, especially on the challenging dataset CaliforniaND which contains various spatial transformations of the identical objects within ND pairs and the visually similar NND pairs. In the future, to deal with the insufficient training data and further improve the performance, we will study weakly supervised learning methods and plan to use other more powerful network structures.

## Figures and Tables

**Table 1:** Channels parameter setting for ST modules in VGG16 and ResNet34.

Backbones	ST Layers	ST-1	ST-2	ST-3
VGG16	localization	64→128→128	128→256→256	256→512→512
	FC	128×24×24→128→32→6	256×10×10→128→32→6	512×3×3→128→32→6
ResNet34	localization	64→128→128	64→128→128	128→256→256
	FC	128×24×24→128→32→6	128×24×24→128→32→6	256×10×10→128→32→6

**Table 2:** Area under the receiver operating characteristic (AUROC) comparison on single modality datasets of ST-CCNN and other state-of-the-art methods.

Methods	CaliforniaND	MFND	
		MFND-IND	MFND-ALL
2-channel-VGG16 [39]	0.792	0.906	0.850
2-channel-ResNet34 [39]	0.852	0.959	0.896
SP-VGG16-PL [2]	0.915	0.914	0.886
SP-VGG16-HY [2]	0.903	0.934	0.910
SP-VGG19-IN [2]	0.887	0.940	0.908
ResNet101-IN [2]	0.936	0.965	0.943
ResNet512-IN [2]	0.927	0.967	0.946
DeepRet500 [2]	0.923	0.994	0.981
DeepRet800 [2]	0.934	**0.996**	0.984
ST-CCNN-VGG16	0.953	0.981	0.955
ST-CCNN-ResNet34	**0.990**	0.994	**0.992**

**Table 3:** AUROC comparison on cross-modality dataset TNO of ST-CCNN and other state-of-the-art methods.

Methods	AUROC
2-channel-VGG16 [39]	0.633
2-channel-ResNet34 [39]	0.663
SP-VGG16-IN [2]	0.679
SP-VGG19-IN [2]	0.667
ResNet101-IN [2]	0.693
ResNet512-IN [2]	0.630
DeepRet-IN [2]	0.745
ST-CCNN-VGG16	**0.764**
ST-CCNN-ResNet34	**0.782**

**Table 4:** Ablation study of with/without triple stream on single modality dataset CaliforniaND.

Models	Backbones	Precision	Recall	F1-Score	AUROC
CCNN-o-cross	(VGG16)	0.811	0.910	0.858	0.910
CCNN-w-cross	(VGG16)	0.826	**0.921**	0.871	0.938
CCNN-o-cross	(ResNet34)	0.820	0.915	0.865	0.935
CCNN-w-cross	(ResNet34)	**0.925**	0.899	**0.912**	**0.959**

**Table 5:** Ablation study of with/without triple stream on cross-modality dataset TNO.

Models	Backbones	Precision	Recall	F1-Score	AUROC
CCNN-o-cross	(VGG16)	0.730	0.748	0.739	0.703
CCNN-w-cross	(VGG16)	0.740	0.763	0.752	0.711
CCNN-o-cross	(ResNet34)	0.715	0.772	0.742	0.710
CCNN-w-cross	(ResNet34)	**0.766**	**0.772**	**0.769**	**0.752**

**Table 6:** Ablation study of with/without ST on single modality datasets CaliforniaND.

Models	Backbones	Precision	Recall	F1-Score	AUROC
CCNN	(VGG16)	0.826	0.921	0.871	0.938
ST-CCNN	(VGG16)	0.928	0.930	0.929	0.953
CCNN	(ResNet34)	0.925	0.899	0.912	0.959
ST-CCNN	(ResNet34)	**0.975**	**0.943**	**0.958**	**0.990**

**Table 7:** Ablation study of with/without ST on single modality datasets TNO.

Models	Backbones	Precision	Recall	F1-Score	AUROC
CCNN	(VGG16)	0.740	0.763	0.752	0.711
ST-CCNN	(VGG16)	**0.805**	0.716	0.758	0.764
CCNN	(ResNet34)	0.766	0.772	0.769	0.752
ST-CCNN	(ResNet34)	0.786	**0.780**	**0.783**	**0.782**

## Data Availability

All real world images appearing in the manuscript are from open access databases.

## Abbreviations

The following abbreviations are used in this manuscript:

CNN	convolutional neural networks
ST	spatial transformer
ST-CCNN	spatial transformer comparing CNN
SIFT	scale-invariant feature transform
HOG	histograms of oriented gradients
VLAD	vector of locally aggregated descriptors
PCA-SIFT	principal component analysis scale-invariant feature transform
LSH	locality-sensitive hashing
BoW	bag-of-word
ROI	regions of interest
ARG	attributed relational graph
GVP	geometry-preserving visual phrases
MOP	multi-scale orderless pooling
MOF	multi-layer orderless fusion
CNNH	convolutional neural network hashing
PCA	principle components analysis
SPoC	sum-pooled convolutional
RPN	region proposal network
ND	near duplicate
NND	non-near duplicate
TP	true positive
TN	true negative
FP	false positive
FN	false negative
MFND	mir-Flickr near duplicate
ROC	receiver operating characteristic
AUC	Area Under Curve
AUROC	area under receiver operating characteristic
